# Supplementary material for: Understanding the impact of urban exposure on obesity among middle and old-age migrants in India
Source: PLoS One. 2025 Jul 30;20(7):e0326096. doi: 10.1371/journal.pone.0326096 (PMC12310033; doi:10.1371/journal.pone.0326096)
Supplement: S1 Appendix — (DOCX) [file pone.0326096.s001.docx]

Supporting information file

| **Variable** | **VIF** | **SQRT VIF Tolerance** | **R-Squared** |
| --- | --- | --- | --- |
| Migration | 1.22 | 1.10 0.8202 | 0.1798 |
| Age | 1.19 | 1.09 0.8436 | 0.1564 |
| Sex | 1.83 | 1.35 0.5472 | 0.4528 |
| Education | 1.29 | 1.14 0.7731 | 0.2269 |
| Work | 1.59 | 1.26 0.6299 | 0.3701 |
| MPCE quantile | 1.09 | 1.04 0.9191 | 0.0809 |
| Caste | 1.21 | 1.10 0.8299 | 0.1701 |
| Religion | 1.11 | 1.05 0.9033 | 0.0967 |
| Physical activity | 1.09 | 1.05 0.9149 | 0.0851 |
| Alcohol consumption | 1.19 | 1.09 0.8399 | 0.1601 |
| Tobacco consumption | 1.16 | 1.08 0.8610 | 0.139 |
| Self-rated health | 1.16 | 1.08 0.8586 | 0.1414 |
| ADL limitation | 1.09 | 1.04 0.9158 | 0.0842 |
| Mobility limitation | 1.21 | 1.10 0.8284 | 0.1716 |
| Sleep problem | 1.04 | 1.02 0.9588 | 0.0412 |
| **Mean VIF** | **1.23** |  |  |
