# Supplementary figures and images for: Understanding the impact of urban exposure on obesity among middle and old-age migrants in India
Source: PLoS One. 2025 Jul 30;20(7):e0326096. doi: 10.1371/journal.pone.0326096 (PMC12310033; doi:10.1371/journal.pone.0326096)

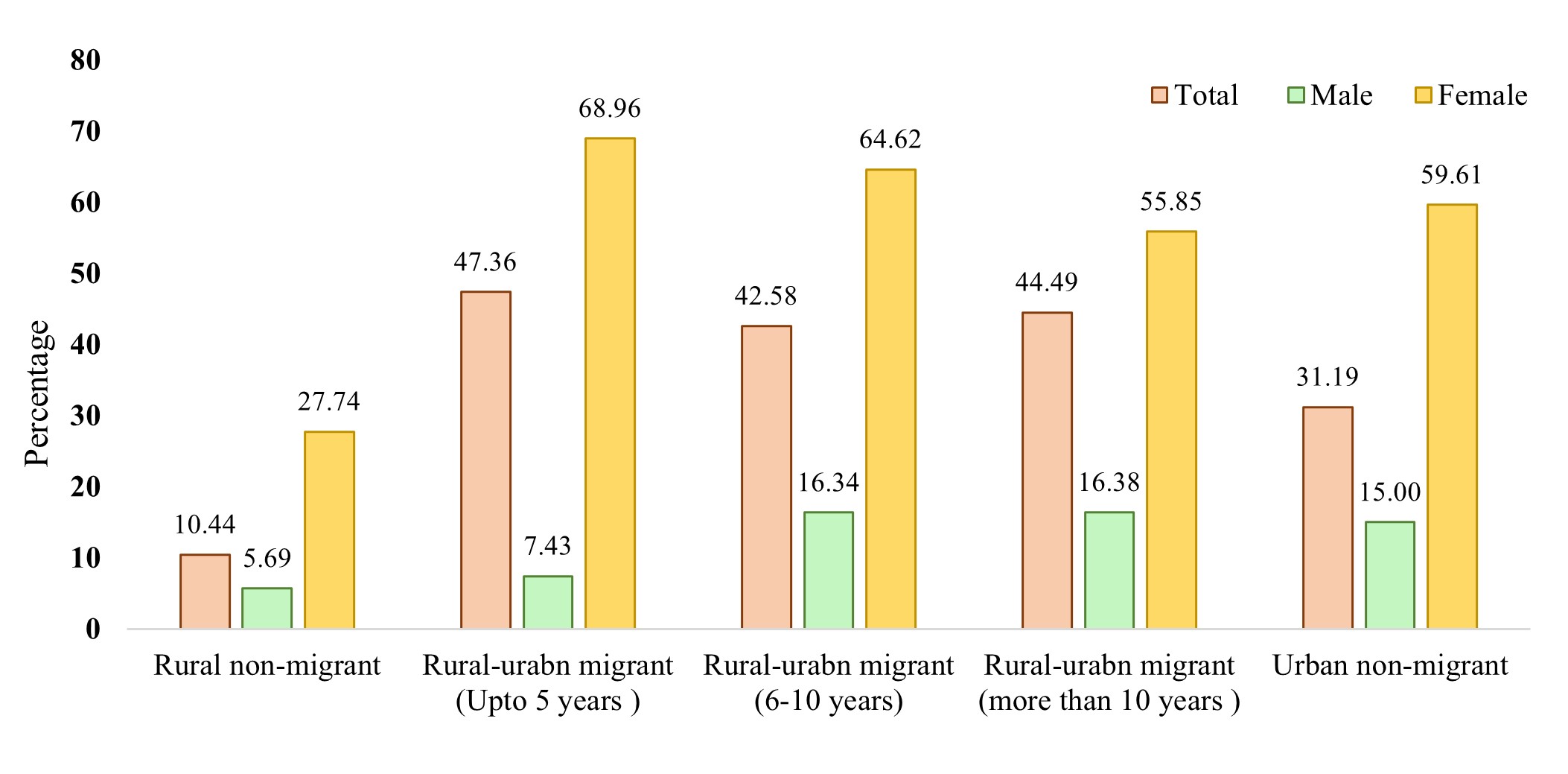

Supplement: S1 File — Figure S1 Kernel destiny distribution of people with waist circumference score stratified by Migration status among the middle-aged and older adults (aged 45+) in India, LASI wave 1 (2017−18). Figure S2 Sex stratified weighted prevalence of people with abdominal obesity by residence status (non-migrants) and duration of residence in urban areas among the middle-aged and older adults (aged 45+) migrants in India, LASI Wave 1 (2017−18). Figure S3 Sex stratified weighted prevalence of people with underweight by migration status among the middle-aged and older adults (aged 45+) in India, LASI Wave 1 (2017−18). Figure S4 Sex stratified weighted prevalence of people with high waist-to-hip ratio by migration status among the middle aged and older adults (aged 45+) in India, LASI Wave 1 (2017−18). Figure S5 Selection criteria of the study sample. (ZIP) [file pone.0326096.s002.zip › SUPPLIMENTARY FIGURES/Supporting Information Figure S2.jpg]

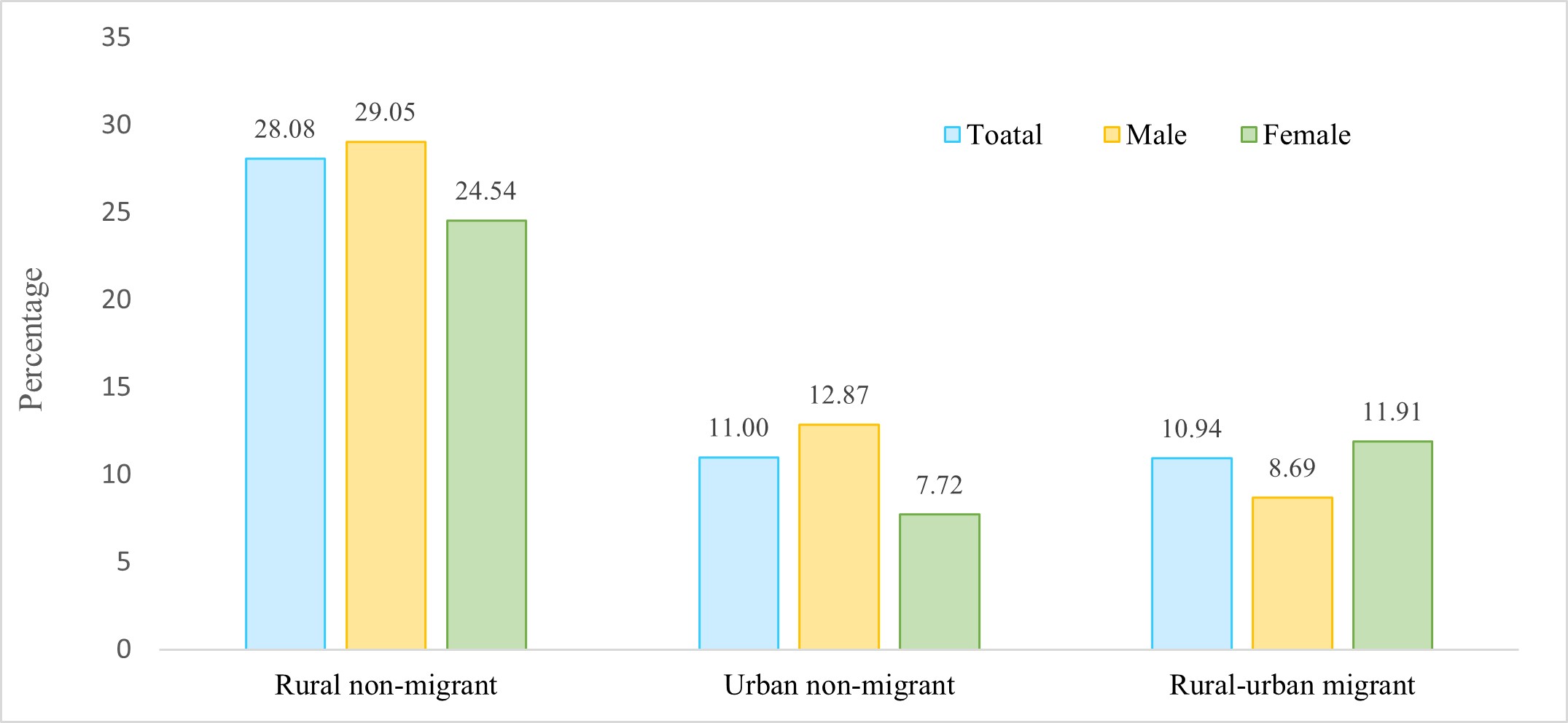

Supplement: S1 File — Figure S1 Kernel destiny distribution of people with waist circumference score stratified by Migration status among the middle-aged and older adults (aged 45+) in India, LASI wave 1 (2017−18). Figure S2 Sex stratified weighted prevalence of people with abdominal obesity by residence status (non-migrants) and duration of residence in urban areas among the middle-aged and older adults (aged 45+) migrants in India, LASI Wave 1 (2017−18). Figure S3 Sex stratified weighted prevalence of people with underweight by migration status among the middle-aged and older adults (aged 45+) in India, LASI Wave 1 (2017−18). Figure S4 Sex stratified weighted prevalence of people with high waist-to-hip ratio by migration status among the middle aged and older adults (aged 45+) in India, LASI Wave 1 (2017−18). Figure S5 Selection criteria of the study sample. (ZIP) [file pone.0326096.s002.zip › SUPPLIMENTARY FIGURES/Supporting Information Figure S3.jpg]

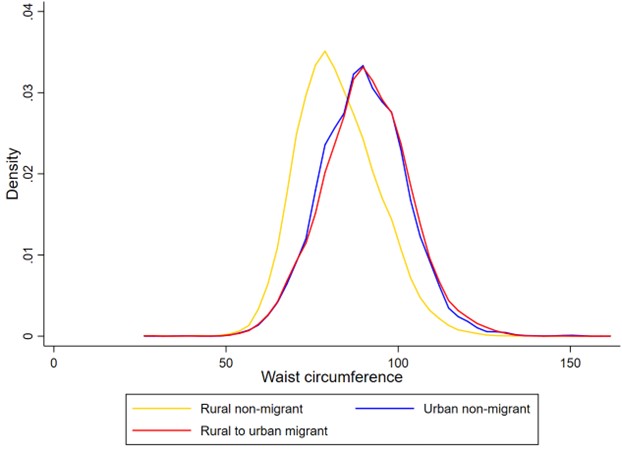

Supplement: S1 File — Figure S1 Kernel destiny distribution of people with waist circumference score stratified by Migration status among the middle-aged and older adults (aged 45+) in India, LASI wave 1 (2017−18). Figure S2 Sex stratified weighted prevalence of people with abdominal obesity by residence status (non-migrants) and duration of residence in urban areas among the middle-aged and older adults (aged 45+) migrants in India, LASI Wave 1 (2017−18). Figure S3 Sex stratified weighted prevalence of people with underweight by migration status among the middle-aged and older adults (aged 45+) in India, LASI Wave 1 (2017−18). Figure S4 Sex stratified weighted prevalence of people with high waist-to-hip ratio by migration status among the middle aged and older adults (aged 45+) in India, LASI Wave 1 (2017−18). Figure S5 Selection criteria of the study sample. (ZIP) [file pone.0326096.s002.zip › SUPPLIMENTARY FIGURES/Supporting Information Figure S1.jpg]

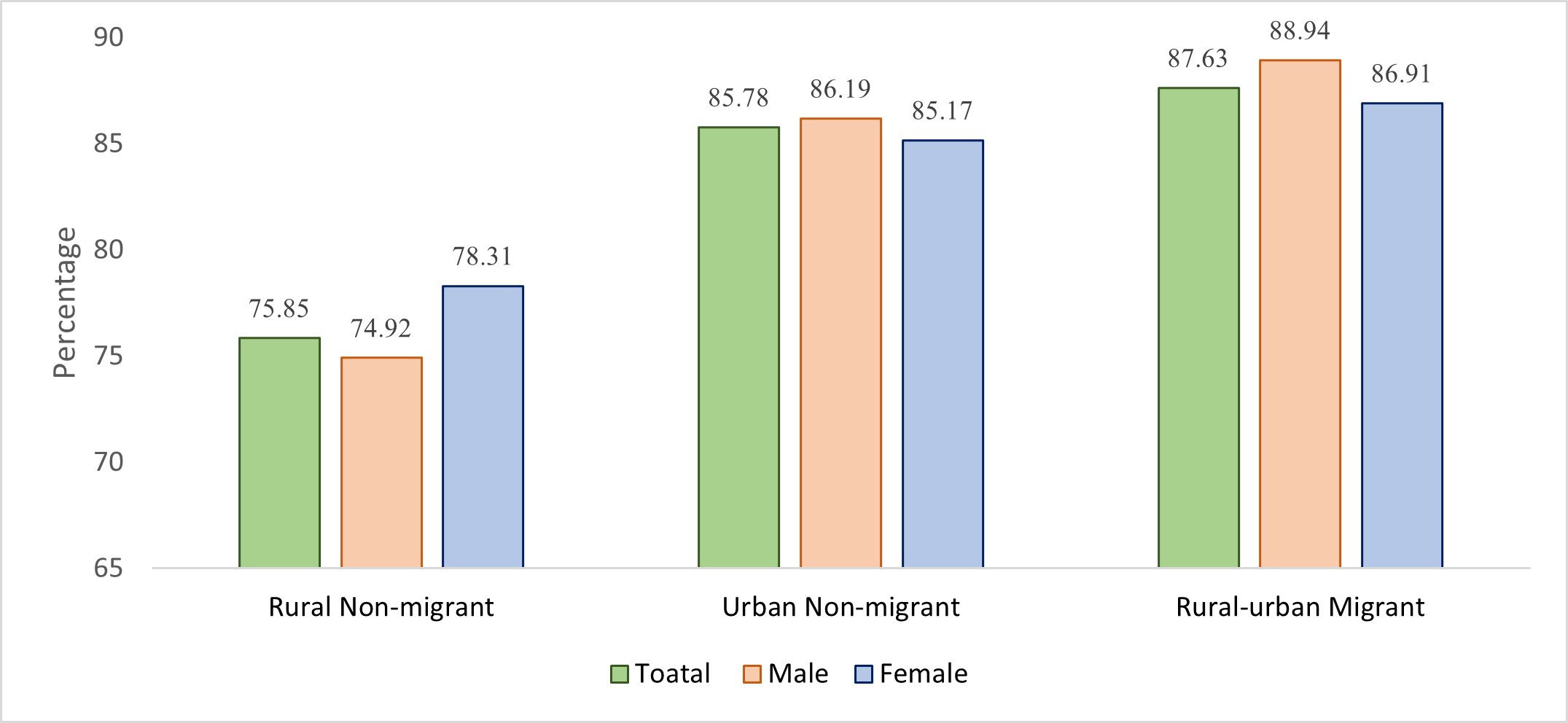

Supplement: S1 File — Figure S1 Kernel destiny distribution of people with waist circumference score stratified by Migration status among the middle-aged and older adults (aged 45+) in India, LASI wave 1 (2017−18). Figure S2 Sex stratified weighted prevalence of people with abdominal obesity by residence status (non-migrants) and duration of residence in urban areas among the middle-aged and older adults (aged 45+) migrants in India, LASI Wave 1 (2017−18). Figure S3 Sex stratified weighted prevalence of people with underweight by migration status among the middle-aged and older adults (aged 45+) in India, LASI Wave 1 (2017−18). Figure S4 Sex stratified weighted prevalence of people with high waist-to-hip ratio by migration status among the middle aged and older adults (aged 45+) in India, LASI Wave 1 (2017−18). Figure S5 Selection criteria of the study sample. (ZIP) [file pone.0326096.s002.zip › SUPPLIMENTARY FIGURES/Supporting Information Figure S4.jpg]

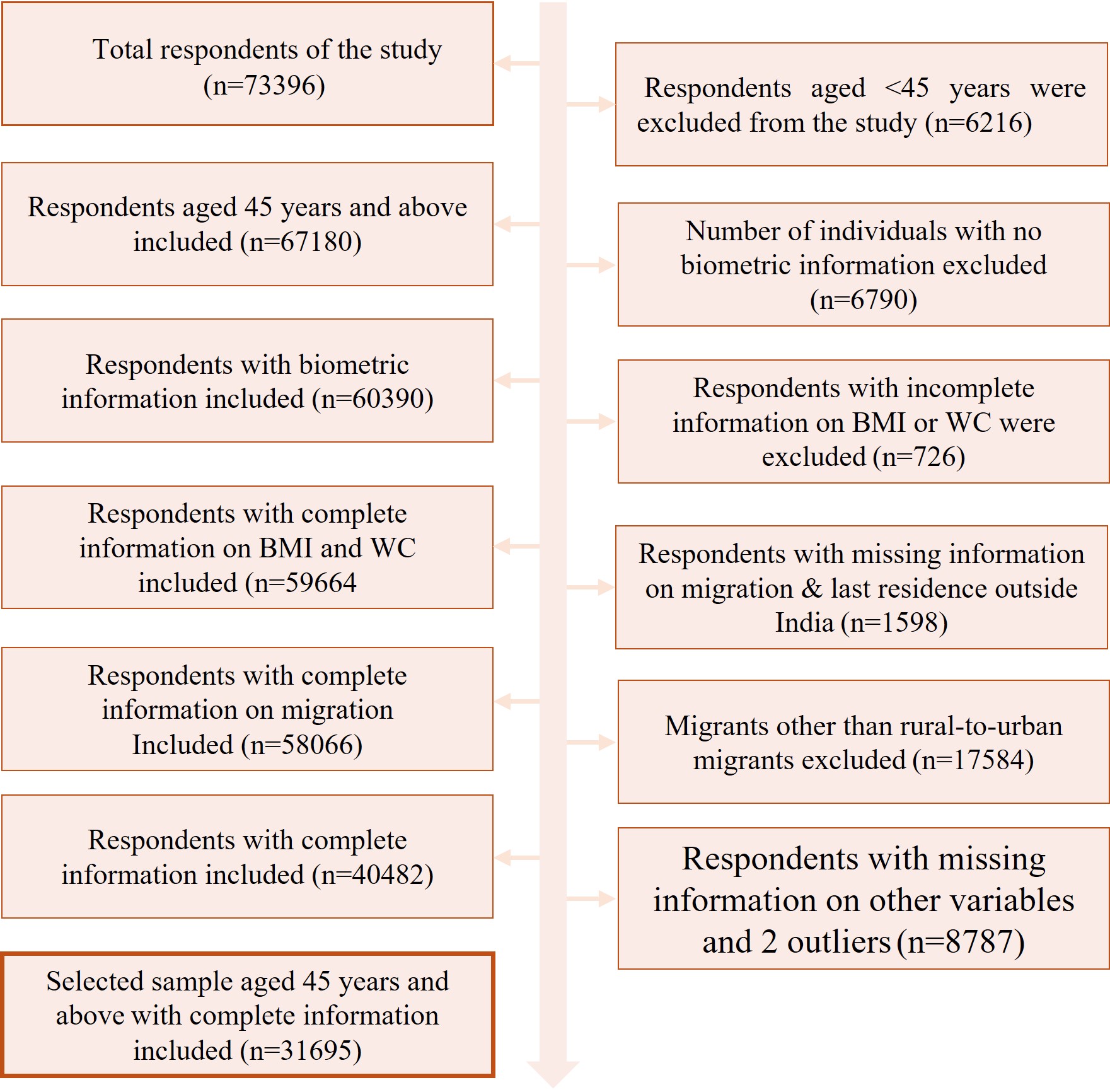

Supplement: S1 File — Figure S1 Kernel destiny distribution of people with waist circumference score stratified by Migration status among the middle-aged and older adults (aged 45+) in India, LASI wave 1 (2017−18). Figure S2 Sex stratified weighted prevalence of people with abdominal obesity by residence status (non-migrants) and duration of residence in urban areas among the middle-aged and older adults (aged 45+) migrants in India, LASI Wave 1 (2017−18). Figure S3 Sex stratified weighted prevalence of people with underweight by migration status among the middle-aged and older adults (aged 45+) in India, LASI Wave 1 (2017−18). Figure S4 Sex stratified weighted prevalence of people with high waist-to-hip ratio by migration status among the middle aged and older adults (aged 45+) in India, LASI Wave 1 (2017−18). Figure S5 Selection criteria of the study sample. (ZIP) [file pone.0326096.s002.zip › SUPPLIMENTARY FIGURES/Supporting Information Figure S5.jpg]
