# Supplementary material for: Understanding the impact of urban exposure on obesity among middle and old-age migrants in India
Source: PLoS One. 2025 Jul 30;20(7):e0326096. doi: 10.1371/journal.pone.0326096 (PMC12310033; doi:10.1371/journal.pone.0326096)
Supplement: Attachment — Submitted filename: Comments.docx [file pone.0326096.s003.docx]

The study addresses a critical gap in research on rural-to-urban migration and its association with obesity in the Indian context, a topic that has been underexplored compared to Western settings. The clear association between prolonged urban residence and elevated obesity risk offers valuable insights into how lifestyle and environmental changes in urban areas contribute to obesity among migrants.

I have few suggestions which is given below:

**Introduction**

The introduction provides a broad overview of the association between migration and health outcomes, specifically obesity. However, the structure could benefit from more logical flow. For example, after discussing the global relevance of rural-to-urban migration, the author could transition more smoothly into the context of obesity in India. Introducing the knowledge gap earlier in the paragraph would make the problem clearer upfront.

Overall, the introduction provides a solid foundation for the study, highlighting the global and Indian context of migration and obesity. However, it could be made more concise, logically organized, and focused on the specific research gap in India.

- Reorganize the introduction to first establish the global connection between migration and obesity, then introduce the Indian context and the gaps in research.

**Methods**

Methods have been clearly explained by the author.

**Results**

Results are well written with full details which will be helpful to the readers.

**Discussion**

- The lack of dietary data is a significant limitation that affects the interpretation of the findings. While the study acknowledges this gap, future research should incorporate dietary assessments to provide a more complete picture of how urban diets contribute to obesity. This could involve collecting detailed information on food consumption patterns and their relationship with obesity.
- The discussion on socio-economic status and its impact on obesity could be expanded.
- The observation of greater obesity risk among female migrants is an important finding. It would be helpful to explain deeper into potential reasons for this disparity, such as cultural, socio-economic, or behavioral factors that might contribute to higher obesity rates in female migrants compared to their male counterparts.
- The manuscript notes that educational attainment did not show a uniform increase in obesity risk. This point could be elaborated further.

Overall, the study provides valuable insights into the relationship between migration and obesity. Addressing these comments and suggestions could further enhance the impact and clarity of the research.
