# Supplementary material for: Understanding the impact of urban exposure on obesity among middle and old-age migrants in India
Source: PLoS One. 2025 Jul 30;20(7):e0326096. doi: 10.1371/journal.pone.0326096 (PMC12310033; doi:10.1371/journal.pone.0326096)
Supplement: Attachment — Submitted filename: R2R.docx [file pone.0326096.s005.docx]

Dear editor,

I would like to express my sincere gratitude for the thorough and insightful guidance provided during the review process of our manuscript. Your expertise and thoughtful suggestions have been instrumental in enhancing the quality and clarity of our work. We deeply appreciate the time and effort you have dedicated to ensuring our research meets the highest standards of publication.
The comments from the reviewers have significantly benefited the manuscript, and we have addressed them to the best of our capacity. We deeply appreciate the time and effort you and the reviewers have dedicated to ensuring our research meets the highest standards of publication.

Journal Requirements:

1. When submitting your revision, we need you to address these additional requirements.

Please ensure that your manuscript meets PLOS ONE's style requirements, including those for file naming. The PLOS ONE style templates can be found at

<https://journals.plos.org/plosone/s/file?id=wjVg/PLOSOne_formatting_sample_main_body.pdf> and <https://journals.plos.org/plosone/s/file?id=ba62/PLOSOne_formatting_sample_title_authors_affiliations.pdf>

- We have thoroughly reviewed and revised our manuscript to ensure it meets all the PLOS ONE style requirements. We have carefully followed the style templates provided and adhered to the file naming conventions as specified by the journal. We believe these adjustments will facilitate a smooth review and publication process. Thank you for guiding us to make these necessary changes.

2. Thank you for uploading your study's underlying data set. Unfortunately, the repository you have noted in your Data Availability statement does not qualify as an acceptable data repository according to PLOS's standards.

At this time, please upload the minimal data set necessary to replicate your study's findings to a stable, public repository (such as figshare or Dryad) and provide us with the relevant URLs, DOIs, or accession numbers that may be used to access these data. For a list of recommended repositories and additional information on PLOS standards for data deposition, please

see <https://journals.plos.org/plosone/s/recommended-repositories>.

- A proper repository of the dataset has now been added. We also shared the replicable data through figshare.

3. In the online submission form, you indicated that your data will be submitted to a repository upon acceptance.  We strongly recommend all authors deposit their data before acceptance, as the process can be lengthy and hold up publication timelines. Please note that, though access restrictions are acceptable now, your entire minimal  dataset will need to be made freely accessible if your manuscript is accepted for publication. This policy applies to all data except where public deposition would breach compliance with the protocol approved by your research ethics board. If you are unable to adhere to our open data policy, please kindly revise your statement to explain your reasoning and we will seek the editor's input on an exemption.

- The data is already available in a public repository. We believe, earlier there was a mistake on our part. We now confirm the accessibility of the data through the publicly available repository

4. Please include your full ethics statement in the ‘Methods’ section of your manuscript file. In your statement, please include the full name of the IRB or ethics committee who approved or waived your study, as well as whether or not you obtained informed written or verbal consent. If consent was waived for your study, please include this information in your statement as well.

- Done!

5. We notice that your supplementary figures are uploaded with the file type 'Figure'. Please amend the file type to 'Supporting Information'. Please ensure that each Supporting Information file has a legend listed in the manuscript after the references list.

- Done!

6. We notice that your supplementary figures and tables are included in the manuscript file. Please remove them and upload them with the file type 'Supporting Information'. Please ensure that each Supporting Information file has a legend listed in the manuscript after the references list.

- Done!

7. Please include captions for your Supporting Information files at the end of your manuscript, and update any in-text citations to match accordingly. Please see our Supporting Information guidelines for more information: <http://journals.plos.org/plosone/s/supporting-information>.

- Done!

8. Please review your reference list to ensure that it is complete and correct. If you have cited papers that have been retracted, please include the rationale for doing so in the manuscript text, or remove these references and replace them with relevant current references. Any changes to the reference list should be mentioned in the rebuttal letter that accompanies your revised manuscript. If you need to cite a retracted article, indicate the article’s retracted status in the References list and also include a citation and full reference for the retraction notice.

- Done!

**Comments to the Author**

**Reviewer 1**

This article deals with a very interesting topic. The relevance of this article is undeniable. Additionally, the article is well structured, the methods of analysis are relevant. However, before its publication, some changes and additions need to be made.

You will find my comments below.

- Thank you for your thoughtful and constructive feedback on our manuscript. We appreciate your recognition of the relevance and structure of our article, as well as the validation of our analytical methods. We are committed to addressing the changes and additions you have suggested to enhance the quality and impact of our work.

1. In the results section, I suggest that the authors present the socio-demographic characteristics of the respondents. It allows to better understand bivariate and multivariate analysis.

- Table of the socio-demographic characteristics of the respondents has been now moved from supplementary to the main manuscript. We briefly discussed the characteristics of the individuals.

2. In the discussion section, I suggest that the authors focus on India, taking into account the socio-demographic characteristics of the country's urban/rural areas. Yes, it's good to compare your results with those of other authors, but the discussion must take account of the country's socio-economic reality.

- Done positively.

3. I suggest that the authors rephrase it ‘This is due to their increased likelihood of acculturation in the urban areas.’ the term acculturation is a bit pejorative. preferably, they have a better chance of adapting to the urban lifestyle and practices.

- Done!

4. I suggest that the authors add a section ‘Policy implication of findings’.

- A new section has now been added before the conclusion, following your instruction.

**Reviewer 2**

The study addresses a critical gap in research on rural-to-urban migration and its association with obesity in the Indian context, a topic that has been underexplored compared to Western settings. The clear association between prolonged urban residence and elevated obesity risk offers valuable insights into how lifestyle and environmental changes in urban areas contribute to obesity among migrants.

- Thank you for your encouraging comments and for recognizing the importance of our study addressing the critical gap in research on rural-to-urban migration and its association with obesity in the Indian context. We are grateful for your acknowledgment of the value our findings.

I have few suggestions which is given below:

Introduction
The introduction provides a broad overview of the association between migration and health outcomes, specifically obesity. However, the structure could benefit from more logical flow. For example, after discussing the global relevance of rural-to-urban migration, the author could transition more smoothly into the context of obesity in India. Introducing the knowledge gap earlier in the paragraph would make the problem clearer upfront.

Overall, the introduction provides a solid foundation for the study, highlighting the global and Indian context of migration and obesity. However, it could be made more concise, logically organized, and focused on the specific research gap in India.-Reorganize the introduction to first establish the global connection between migration and obesity, then introduce the Indian context and the gaps in research.

- Thank you for your constructive critique regarding the structure of our introduction. We have considered your feedback and revised it accordingly, enhancing the logical flow by clearly linking the global context of migration to the specific issues of obesity in India early in the text.

Methods
Methods have been clearly explained by the author.

Results
Results are well written with full details which will be helpful to the readers.
Discussion
-The lack of dietary data is a significant limitation that affects the interpretation of the findings. While the study acknowledges this gap, future research should incorporate dietary assessments to provide a more complete picture of how urban diets contribute to obesity. This could involve collecting detailed information on food consumption patterns and their relationship with obesity.

The discussion on socio-economic status and its impact on obesity could be expanded.

- We have addressed this positively.

The observation of greater obesity risk among female migrants is an important finding. It would be helpful to explain deeper into potential reasons for this disparity, such as cultural, socio-economic, or behavioral factors that might contribute to higher obesity rates in female migrants compared to their male counterparts.

- Explanation has been added following your suggestion.

The manuscript notes that educational attainment did not show a uniform increase in obesity risk. This point could be elaborated further. Overall, the study provides valuable insights into the relationship between migration and obesity. Addressing these comments and suggestions could further enhance the impact and clarity of the research.

- Done!
