# Supplementary material for: Understanding the impact of urban exposure on obesity among middle and old-age migrants in India
Source: PLoS One. 2025 Jul 30;20(7):e0326096. doi: 10.1371/journal.pone.0326096 (PMC12310033; doi:10.1371/journal.pone.0326096)
Supplement: Attachment — Submitted filename: Responce to reviewer.docx [file pone.0326096.s007.docx]

Review Comments to the Author

Please use the space provided to explain your answers to the questions above. You may also include additional comments for the author, including concerns about dual publication, research ethics, or publication ethics. (Please upload your review as an attachment if it exceeds 20,000 characters)x

Reviewer #3: Technical Soundness and Data Support for Conclusions
Strengths: The study is well-designed, using a nationally representative dataset (LASI) and robust methods (logistic/quantile regression). The conclusions align with global literature on migration and obesity.

Limitations: The cross-sectional design limits causal inference. Lack of dietary data is noted, but the authors justify this by referencing prior studies on urban dietary shifts. Statistical Analysis
Appropriateness: The use of logistic regression (for binary outcomes) and quantile regression (to explore BMI distribution) is rigorous. Multicollinearity checks (VIF < 2) and sensitivity analyses (waist circumference, waist-hip ratio) strengthen validity.

Suggestions: Clarify why certain covariates (e.g., alcohol/tobacco) showed inconsistent associations. Additional robustness checks (e.g., stratified analyses by gender/age) could enhance insights.

- *In response to your suggestion, we have now included stratified analyses by gender and age group to provide more robust evidence on the migration–obesity relationship.*

*We also acknowledge the observed inconsistencies in the association between alcohol/tobacco consumption and obesity. We have elaborated on the potential explanations for these patterns.*

*In the Indian context, particularly among older adults in rural and traditional settings, alcohol and tobacco use are socially sensitive topics. These behaviours are often considered taboo, especially among women and the elderly, leading to substantial underreporting due to fear of social judgment or stigma. Given that our study relied on self-reported data, this introduces a significant risk of misclassification bias. Participants who consume alcohol or tobacco may have reported themselves as non-users, or non-frequent (or heavy) consumers of alcohol, thereby weakening or distorting the observed associations with obesity. This reporting bias likely contributed to the inconsistency of associations in our findings.*

*Notably, similar insights have been observed in previous studies using LASI data. For example, a recent study by Saha et al. (2023) published in PLOS ONE* [*(https://doi.org/10.1371/journal.pone.0291920)*](https://doi.org/10.1371/journal.pone.0291920) *also found inconsistent associations between alcohol/tobacco use and obesity among older adults, attributing the variation in part to social desirability and reporting biases. This further supports the interpretation that underreporting in culturally sensitive variables may obscure true epidemiological relationships in such datasets*

*Beyond India, a wide body of international literature also highlights that the relationship between alcohol and tobacco use with obesity is not consistent [*[*Link-1*](https://doi.org/10.1186/s12889-023-16946-4) *,* [*Link-2*](https://doi.org/10.3349/ymj.2001.42.5.480)*]. Studies have shown that variability in consumption patterns, such as frequency, quantity, type of substance, and duration, plays a critical role in shaping outcomes. For instance, moderate alcohol consumption may have different metabolic effects than heavy or binge drinking. Similarly, smoking’s impact on weight may differ by intensity and time since cessation. Moreover, quantile regression analyses and similar statistical approaches in other settings have revealed differential effects across the BMI distribution [Please refer to figure 3 in the manuscript]. This suggests that the impact of alcohol or tobacco use may be significant at certain BMI percentiles but negligible at others. Finally, the association is often confounded by other lifestyle behaviours such as diet, or other socioeconomic/ behavioural status. These are factors that may not be fully captured or accurately adjusted for in self-reported survey data. While our models included key covariates, residual confounding remains a plausible contributor to the observed inconsistencies. In sum, both national and global evidence suggest that measurement error, social desirability bias, non-linear associations, and lifestyle confounding collectively contribute to the variable and sometimes contradictory relationship between alcohol or tobacco use and obesity in observational data.*

*Link-1: https://doi.org/10.1186/s12889-023-16946-4*

*Link-2: https://doi.org/10.3349/ymj.2001.42.5.480*

Data Availability

Compliance: Uploaded replicable data to figshare (DOI: 10.6084/m9.figshare.27266337.v1). The primary dataset is publicly available via the Gateway to Global Aging Data ([www.g2aging.org](http://www.g2aging.org)).

Final Assessment

The manuscript is technically sound, with conclusions well-supported by data. Statistical methods are appropriate and rigorous, though minor clarifications could improve transparency. Data availability meets PLOS ONE standards after revision.

The Conclusion section of the manuscript does not clearly and directly address the study's stated objectives. Here’s a breakdown of the issues and suggestions for improvement: Key Problems with the Current Conclusion
Lacks Direct Link to Objectives

The study aimed to:
Examine the impact of rural-to-urban migration on obesity.
Compare obesity risk by duration of urban residence.
The conclusion vaguely summarizes findings but does not explicitly tie them back to these objectives.
Too General and Repetitive
It reiterates results (e.g., "prolonged urban residence is progressively associated with obesity risks") without synthesizing implications.

- *Thank you for the valuable feedback. We have now revised the Conclusion section to clearly and directly address the study's stated objectives. The revised conclusion explicitly links the findings to the aims of examining the impact of rural-to-urban migration on obesity and comparing obesity risk by duration of urban residence. We have also refined the language to avoid repetition and have included a more focused synthesis of the study’s key implications.*

Reviewer #4: The manuscript provides a comprehensive analysis of the association between rural-to-urban migration and obesity among middle-aged and older adults in India. The study is well-structured, methodologically sound, and addresses an important public health issue. However, some areas require clarification, refinement, or expansion to enhance the manuscript's impact and readability:

- Clarify the Research Gap: The introduction highlights the lack of nationally representative studies on migration and obesity in India but could better emphasize the novelty of focusing on middle-aged and older adults.

- *Thank you for the insightful comment. In the revised manuscript, we have addressed this by further clarifying the research gap in the Introduction section. We now place greater emphasis on the novelty of focusing specifically on middle-aged and older adults.*

- Migration Definition: Line 143-146: Based on the definition, it seem like even participants who just moved this area for 1 day were also categorized as migrant. Is there any reference to support this?

- *Thank you for the comment. In the revised manuscript, we have clarified the definition of migration by detailing the criteria used to identify migrants. We have also included supporting references to show that this approach, has been adopted in previous studies using the same dataset.*

Please verify some typo errors:
- Line 380: Socio-economic stata --> Socioeconomic status

- *Done!*

Based on the reviewers’ observations and considering the significance of the tables in supporting the key findings, we have now moved some of the tables from the supplementary section to the main manuscript. This has been done to present the results more clearly and to provide stronger and more direct support to the study's conclusions.
